# What Is the Most Realistic Single-Compartment Model of Spike Initiation?

**DOI:** 10.1371/journal.pcbi.1004114

**Published:** 2015-04-09

**Authors:** Romain Brette

**Affiliations:** 1 Institut d’Etudes de la Cognition, Ecole Normale Supérieure, Paris, France; 2 Sorbonne Universités, UPMC Univ. Paris 06, UMR_S 968, Institut de la Vision, Paris, France; 3 INSERM, U968, Paris, France; 4 CNRS, UMR_7210, Paris, France; The University of Texas at Austin, United States of America

## Abstract

A large variety of neuron models are used in theoretical and computational neuroscience, and among these, single-compartment models are a popular kind. These models do not explicitly include the dendrites or the axon, and range from the Hodgkin-Huxley (HH) model to various flavors of integrate-and-fire (IF) models. The main classes of models differ in the way spikes are initiated. Which one is the most realistic? Starting with some general epistemological considerations, I show that the notion of realism comes in two dimensions: empirical content (the sort of predictions that a model can produce) and empirical accuracy (whether these predictions are correct). I then examine the realism of the main classes of single-compartment models along these two dimensions, in light of recent experimental evidence.

## Introduction

**Fig 1:**
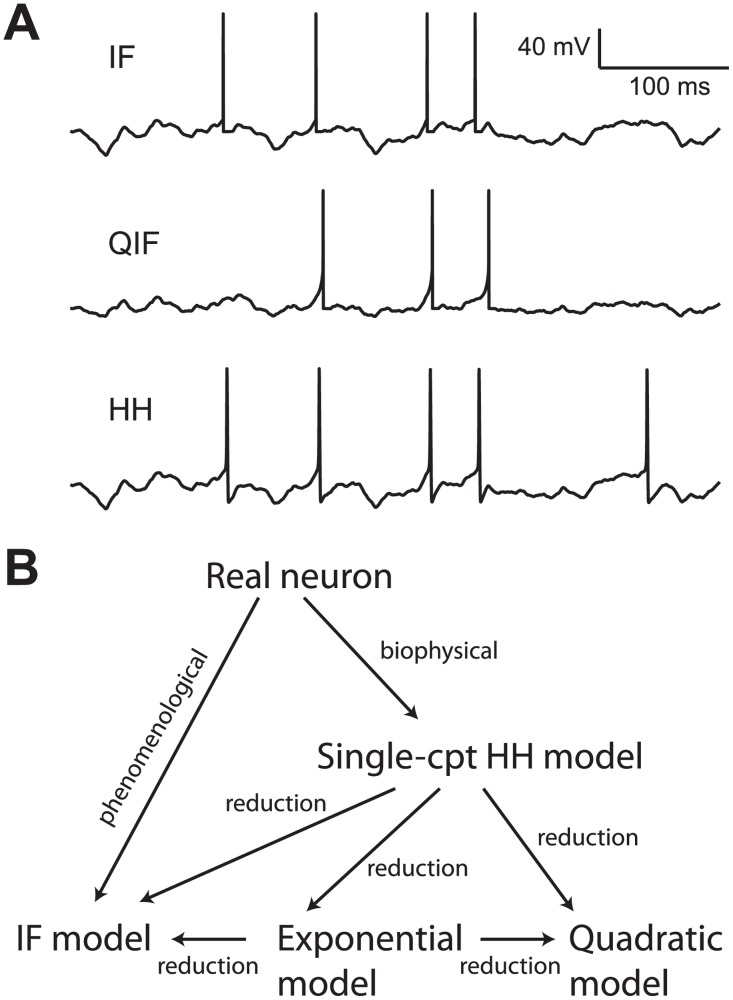
Single-compartment models. (A) Voltage traces of 3 models for the same input current: integrate-and-fire (IF), quadratic (QIF) and Hodgkin-Huxley (HH). The models differ mainly in their spiking responses. (B) Relationships between different single-compartment models. The IF model is a phenomenological model of spike initiation, while the HH model is a biophysical model. Other models can be obtained by reduction, i.e., as approximations of one of the above models under specific conditions.

A large variety of neuron models are used in theoretical and computational neuroscience, ranging from simple integrate-and-fire (IF) models to multicompartmental biophysical models based on Hodgkin-Huxley (HH) theory (Fig. 1) [1]. The rationale behind the choice of model for a given study generally follows the simplicity-realism trade-off: simple enough to be useful, but realistic enough to be meaningful. For this reason, the morphological structure of neurons is ignored in many neuron models. These models are generally called *single-compartment models*, and include neither the dendrites nor the axon. Among these models, there is a wide range of models with various degrees of complexity. A major difference between them is the way spikes are initiated: by definition, in the integrate-and-fire model, spikes are produced when the voltage exceeds a threshold; in the HH model and reduced models (quadratic [2] and exponential [3]), spikes implicitly result from the dynamics of the model. These major differences come in a number of variations, for example with the inclusion of adaptation currents. This review focuses on how these models differ with respect to spike initiation. Which one is most realistic?

## Single-Compartment Models

A single-compartment model is a model that neglects the spatial dimension. There are two ways to justify this choice. One is to consider that the membrane potential and other relevant biophysical quantities are identical everywhere in the neuron. Models following this view are also called *isopotential* models. A possible justification is that some neurons have small dendritic tree—these neurons are called *electrotonically compact* cells—along which voltage attenuation is small. This justification is relatively weak for two reasons. First, voltage attenuation is frequency-dependent: at higher oscillation frequencies, voltage decays on smaller spatial scales [4]. Second, active conductances distributed along the dendrites and axon and even passive synaptic inputs can introduce large voltage variations on short spatial scales [5,6].

Another way to justify the use of a single-compartment model is to consider that the voltage variable represents the membrane potential at one particular location, generally the soma. The model must then be adjusted to compensate for the absence of dendrites and axon and preserve the correct properties of the neuron, which makes it an *effective* or phenomenological model. For example, the membrane time constant and capacitance could be chosen so that the model correctly predicts the neuron’s response to a step current injected at the soma. In this sense, all single-compartment models are phenomenological, including HH models. In the following, I will assume that the single compartment represents the soma, but I will come back to this issue in the discussion.

These models describe both how the membrane potential changes in response to a current, and predict when spikes are emitted. The input current may represent a current injected through an electrode, or the total synaptic current coming from the dendrites and/or soma. There are many such models (Fig. 1A). The reference is the HH model, a biophysical model obtained from patch-clamp measurements of ionic currents passing through voltage-gated channels [7]. It comes in a large number of variations, depending on the nature and properties of ionic channels, but all these variations share the feature that spikes are implicitly generated by the dynamics of the model. In contrast, in the integrate-and-fire model, a spike is explicitly produced when the membrane potential reaches a predefined threshold. The IF model could be seen as an approximation of the HH model [8] or simply as a phenomenological description of spike generation, since the IF model was introduced before biophysical descriptions [9] (Fig. 1B). The IF model is sometimes called the *leaky* integrate-and-fire model, so as to distinguish it from more recent variations. The *quadratic* integrate-and-fire model is dynamically equivalent to type I HH models for constant currents near rheobase [2]. The *exponential* integrate-and-fire model includes an approximation of the sodium (Na) current [3] and is known to be a good approximation of HH models with fluctuating inputs [10]. In these models, spikes are implicitly generated by the dynamics of the model, when the membrane potential diverges to infinity.

Each of these models comes in a number of variations. For example, the Izhikevich model is a quadratic model with an additional adaptation process [11]. The AdEx model is an exponential model with an additional adaptation process [10]. More generally, all types of models can be complemented with various models of ionic currents, depending on the particular neuron being modeled. What distinguishes the different kinds of models is the way spikes are generated: by a sharp voltage threshold in the leaky integrate-and-fire model, by a smooth implicit threshold in the HH model (Na channels open gradually with voltage) [3]. Therefore, the discussion will focus on spike initiation in single-compartment models.

Presenting the models as approximations of the HH model suggests a natural ordering of models by decreasing degree of realism: HH, exponential, quadratic, and IF. However, this intuition could be incorrect, because all these models, including the HH model, are single-compartment models and therefore strongly idealized. I will review the experimental evidence relevant to this question, but because the conclusion will be counterintuitive, we first need to clarify what exactly is meant by “realistic.”

## What Is a “Realistic” Model?

Is a more detailed model necessarily more realistic? Consider two models of a plane: a toy plane made of wood and a simple paper plane. The first model is more detailed, and it has different recognizable elements of a plane: wings, helixes, a cockpit. The second model is more abstract, but it has an important characteristic that the first model does not have: it can fly. From a scientific point of view, the first model is detailed but not realistic, since it cannot fly. Level of detail and scientific realism are two different notions.

The HH model has Na channels while the IF model does not. Of course, the HH model does not literally have ionic channels. It has equations and variables that we name “Na current” or “gating variable,” which may or may not correspond to properties of the actual channels. The HH model has ionic channels in the same sense that the wooden plane has a helix: there is something we call a “helix,” but what it really is is a piece of wood, which may or may not make the plane fly. For example, in the original HH model, the Na current corresponds to something that can be physically measured, but the Na gating variable (m) has no biophysical counterpart in the actual protein. The current is modeled as proportional to m^3^ only because exponent 3 was the best fit to the data. We call it the gating variable only because it is part of a story in which it is a gating variable: the story that there are three independent gates that must all be open for the channel to be open. It is an elegant story, but we now know that this is not what happens with the Na channel [12]. So the structure of the model is consistent with a story in which there is a neuron with Na channels, but this particular story is a fiction. The realism of the HH model is not to be found in the story, but rather in the predictions that it can make.

**Fig 2:**
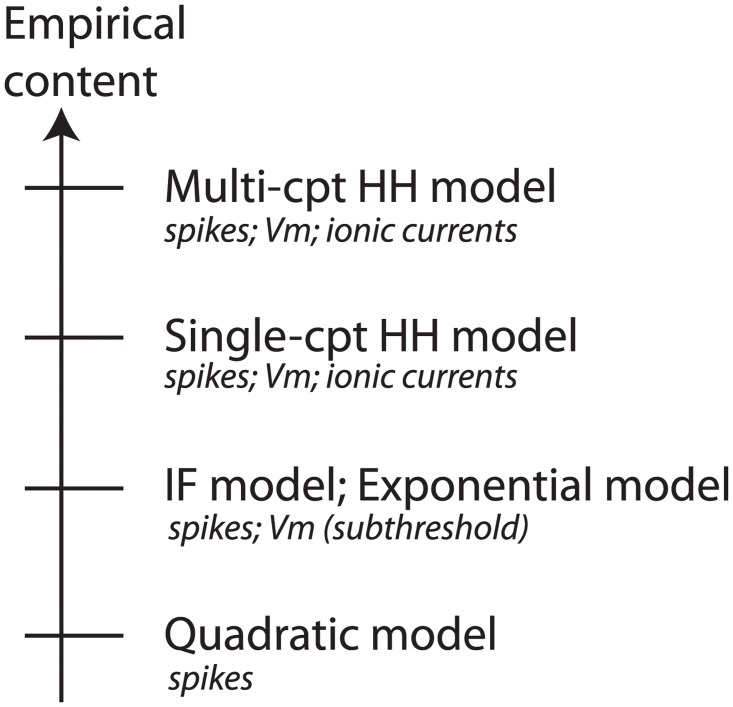
Empirical content of models, from the quadratic model (only spike trains are predicted) to multi-compartmental HH models (Membrane potential and ionic currents are predicted at different locations on the neuron).

Therefore, we must carefully distinguish between stories (“gating variables”) and actual scientific content—that is, the articulation of the model with reality. The added value of detailed models can be comprehended in a more satisfying way using the concept of *empirical content* described by philosopher of science Karl Popper [13]. The empirical content of a theory is the set of possible falsifiers of the theory. In short, for a model, it is the type of predictions that a model can make, which can be falsified. For example, the integrate-and-fire model can make predictions about the membrane potential and the spike times as a function of the input current. The HH model can additionally make predictions about the Na and potassium currents using pharmacological manipulations. The quadratic model can only make predictions about spike times, because the main variable is abstract and has no direct connection with the membrane potential. Fig. 2 shows a number of models ordered by their level of empirical content: the HH model is the single-compartment model with the largest empirical content.

Empirical content only refers to the logical structure of the models, that is, the kind of predictions they can make, but it does not address the question of whether these predictions are accurate or not. Therefore, it cannot be identified with realism. Additionally, one must consider the *empirical accuracy* of models.

## Empirical Accuracy

The accuracy of the original HH model was primarily demonstrated with experiments on a space-clamped squid giant axon: a wire was inserted into the axon so as to short-circuit the longitudinal resistance of the axoplasm [14]. In this configuration, the axon is effectively isopotential and therefore the use of a single-compartment model is appropriate. The model was also validated with respect to spike propagation along the axon (in particular, to predict conduction velocity), but in this case the full cable equation was used, not a single-compartment model. Therefore, it is not entirely clear that the same single-compartment model can adequately account for spike initiation when the neuron is not isopotential, as is the case with many neurons with a large soma that collects dendritic currents and a thin axon where spikes are initiated.

### Additional Empirical Content

Compared with integrate-and-fire models, the HH model has additional empirical content: it can make predictions about spike shape and about ionic currents. How accurate are these additional predictions?

**Fig 3:**
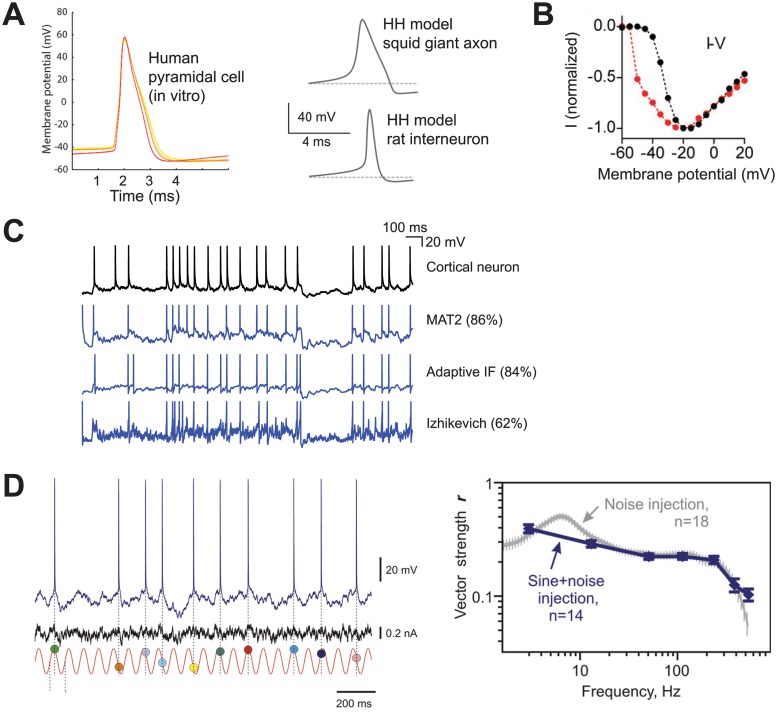
Empirical accuracy of the single-compartment HH model. (A) Spikes of real neurons have a distinct kink at onset (top: human pyramidal cell, adapted from [18]), which does not appear in single-compartment HH models (bottom: HH models of squid giant axon and rat hippocampal interneuron, adapted from [19]). (B) The peak current in somatic voltage-clamp shows a discontinuity as a function of voltage (red), not seen when axonal Na channels are inactivated (black) (adapted from [20]). (C) Spiking responses of cortical neurons in vitro to somatic current injection (black) are well predicted by integrate-and-fire models with sharp spike initiation (blue, first two traces; percentages are proportion of correctly predicted spikes) but not by the Izhikevich model, a variation of the quadratic model (blue, third trace) (adapted from [22]). (D) Phase locking to high frequencies: cortical neurons fire at preferred phases of a sinusoidal current added on top of noise (left), at frequencies up to several hundred Hz (right, vector strength is a measure of phase locking) (adapted from [23]).

Let us start with spike shape. Although the single-compartment HH model qualitatively reproduces the rise and fall of action potentials observed at the soma, which cannot be reproduced by integrate-and-fire models, by definition, it fails at accounting for the shape of spikes near spike initiation (Fig. 3A) [15]. Specifically, these models predict that the onset of spikes should be shallow because Na channels open gradually with voltage: membrane potential must increase by about 12 mV for the proportion of channels to rise from 27% to 73% (twice the Boltzmann activation slope k_a_, which is about 6 mV) [16,17]. In contrast, there is a distinct *kink* at spike onset, which appears in a voltage trace as a rapid voltage transition from the resting membrane potential.

Compared with integrate-and-fire models, the HH model also makes predictions about ionic currents involved in the initiation of spikes, in particular the Na current. This current can be measured in voltage-clamp with pharmacological manipulations (blocking potassium currents). The membrane potential is increased from rest to a target value (step) and the Na current is measured. The HH model predicts that the peak Na current varies gradually with membrane potential. In contrast, in a somatic voltage-clamp, the relationship between membrane potential and peak current shows an abrupt jump above a critical voltage value [20] (Fig. 3B, red). This is a generic finding that was observed not only in cortical neurons, but also in the brainstem and in motoneurons.

The current-voltage relationship can also be measured in somatic current-clamp, in neurons driven by noisy currents [21]. In cortical neurons, it was found that this relationship can be fitted by the sum of a linear function (representing the leak current) and an exponential function (representing the Na current). Single-compartment HH models, constrained by patch-clamp measurements of channel properties, predict that the curvature of the exponential function equals the Boltzmann activation slope k_a_ ≈ 6 mV. Instead, the measured curvature is exceptionally sharp, on the order of 1 mV.

These discrepancies with single-compartment HH models are well known, and their origin has been attributed to currents flowing from the axon at the initiation of spikes [20,24]: indeed, they are not seen when axonal Na channels are inactivated (Fig. 3B, black). However, it remains that single-compartment HH models cannot account for these phenomena. I stress again that I am referring to HH models in the way they are normally used, that is, where channel properties are constrained in some way by patch-clamp measurements. It would be possible to reproduce the sharp onset of somatic spikes and the discontinuous current-voltage relationship by considering that the activation function of Na channels is a step function of membrane potential, effectively making the model an integrate-and-fire model—but this is not usually considered an HH model.

Therefore, the additional empirical content of single-compartment HH models, compared to integrate-and-fire models, is in fact not empirically accurate. However, it could still be that these are more realistic in terms of predicting the spike trains in response to an input current—that is, the common empirical content.

### Common Empirical Content

The exceptional sharpness measured in current-voltage relationships was also found in the same study to be important for predicting the spiking responses of cortical neurons with an exponential model [21]. With a sharpness factor of 1 mV, an exponential model is in fact very close to an IF model. This result belongs to a line of recent studies showing that precise spiking responses of cortical neurons to noisy currents injected at the soma are surprisingly well predicted by adaptive integrate-and-fire models (Fig. 3C) [25].

To this date, there is a single study reporting a successful fit of a single-compartment HH model to in vitro recordings [26]. This scarcity is likely due to the large number of parameters and also to the fact that the formal structure of HH models (type of channels and specific equations) must be known. In that study, performance in predicting spikes seemed quite good (coincidence factor Γ = 0.38 compared to an intrinsic reliability of 0.52). Unfortunately, there was no comparison with integrate-and-fire models, the recorded neurons (zebra finch HVC) have not been used in other fitting studies and the injected current was very different from those used in related studies. However, it is known that the spiking responses of HH models are well fitted by exponential IF models with adaptation [10], and the latter models have been shown to predict spiking responses of cortical neurons with high accuracy [22,27]. But the optimal value for the slope factor (curvature of the exponential function) was found to be 0 mV. This means that the best fitting model was in fact a standard integrate-and-fire model (with adaptation)—although it is possible that performance showed little decrease with higher slope factors. The quadratic integrate-and-fire model with adaptation [11] showed poor prediction performance. A recent in vivo study also showed that spiking can be accurately predicted from membrane potential on the basis of a sharp (but dynamic) threshold [28].

In addition, cortical neurons can reliably transmit frequencies up to several hundred Hz and respond to input changes at the millisecond timescale [23,29]. This means that when a small high frequency sinusoidal current or a small current step is added on top of a background noisy current injected in the cell, the time-varying probability of discharge closely follows the input current (Fig. 3D). This is surprising because according to theoretical studies, the cut-off frequency of signal transmission in single-compartment models is inversely related to the activation slope factor of Na channels [3]. On this basis, the cut-off frequency of single-compartment HH models should be one order of magnitude lower than empirically observed. In contrast, standard IF models can transmit very high frequencies [30]—the precise value of the cut-off frequency might be achieved by an exponential IF model with a small slope factor [3].

In summary, it appears that standard IF models better account for the spiking responses of cortical neurons than single-compartment HH models. Given that the additional empirical content of the latter models is also inaccurate, it appears that the IF model is more realistic than the single-compartment HH model with respect to spike initiation.

## Theoretical Explanations

How is it possible that the IF model, a simple phenomenological description of spike initiation, is more accurate than a biophysical model of spike initiation? Three explanations have been proposed in the literature: the cooperativity hypothesis [15], the active backpropagation hypothesis [24] and the compartmentalization hypothesis [31].

**Fig 4:**
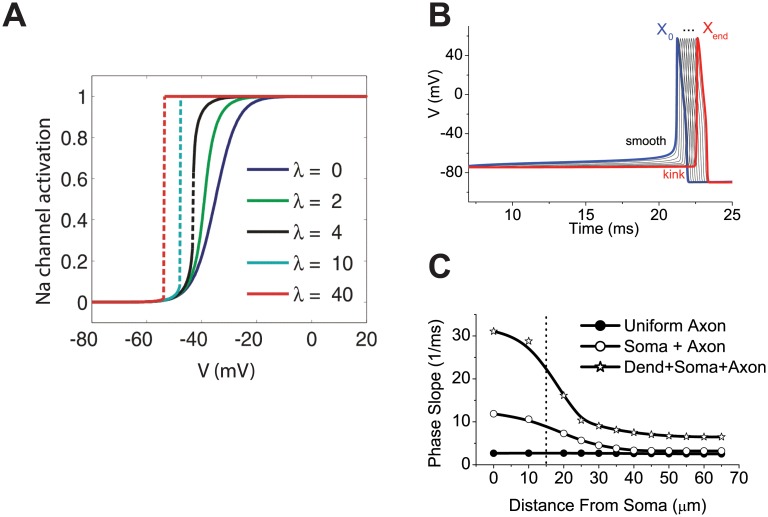
Cooperativity and backpropagation hypotheses. (A) The cooperativity hypothesis (adapted from [40]). Collective activation curve of Na channels as a function of coupling strength λ (0: no coupling). When cooperativity is strong enough, collective activation becomes step-like: Na channels open all at once when a threshold voltage is exceeded. (B and C) The active backpropagation hypothesis (adapted from [39]). (B) When a spike propagates forward along an active 2 mm-long cylinder of 1 μm diameter (model), its onset becomes progressively sharper. (C) Onset rapidness as a function of distance from soma where the spike is measured, for three models all including a 50 μm long axon (1 μm diameter) and a soma: soma with the same diameter as the axon (“Uniform axon”), large soma (“Soma+Axon”), and large soma and dendrites (“Dend+Soma+Axon”).

It was proposed that Na channels cooperate, which would make their collective activation curve much sharper [15]. According to this hypothesis, the activation curve of a single channel shifts to hyperpolarized voltages when neighboring channels open (Fig. 4A, top). As a result, the collective activation curve is sharper, and can even be a step-like function of voltage, meaning that Na channels open all at once when the voltage exceeds a threshold (Fig. 4A, bottom). Cooperative activation has been demonstrated in calcium [32,33], potassium [34] and HCN channels [35], and in pharmacologically altered Na channels of cardiac myocytes [36]. However, in Na channels of the axon initial segment (AIS), where spikes are initiated, this hypothesis is not supported by direct experimental evidence. Voltage-clamp recordings from axonal blebs found no sign of cooperativity [37], although the initial segment could have been damaged by the formation of the bleb [38]. Spikes recorded in current-clamp in axonal blebs also show no kink at onset, while spikes simultaneously recorded in the soma do [24,39], and it is not clear how damage to the AIS would affect onset rapidness at the bleb but not at the soma.

The second explanation relies on standard biophysics but takes into account the spatial extent of neurons. As I previously pointed out, the single-compartment HH model is an idealized model of spike initiation. In particular, it has been known for a long time that in cortical pyramidal cells, spikes are initiated in the AIS, about 20–40 μm from the soma [41–43]. It has been proposed that the kink at spike onset results from active backpropagation (Fig. 4B-C): spikes are initiated in the axon and backpropagated to the soma, so that the kink reflects the sharpened current coming from the axon [24,39], an observation already made in the early days of electrophysiology [44]. In particular, it is possible to replicate the phenomenon in multicompartmental models based on standard HH formalism [37,39]. However, the active backpropagation hypothesis focuses on spike shape and does not explain why spike initiation is sharp as in an IF model. On the contrary, according to this explanation, spikes are initiated as in a single-compartment HH model, where the compartment is the AIS, and only the shape of somatic spikes is modified, not the spiking properties. But this is contradicted by the evidence reviewed above. In addition, sharpening by active propagation is expected to occur on distances greater than the characteristic length of the axon (Fig. 4B shows propagation over 2 mm), but that length is about 500 μm in cortical pyramidal cells while the AIS is only about 30 μm from the soma. Accordingly, as shown by the same authors in simple models, sharpening does not occur over a 50 μm-long axon if the soma has the same diameter as the axon, but it does if the soma is much larger (Fig. 4C). These observations suggest that the phenomenon is related to the size of the soma relative to the axon, rather than to active backpropagation.

**Fig 5:**
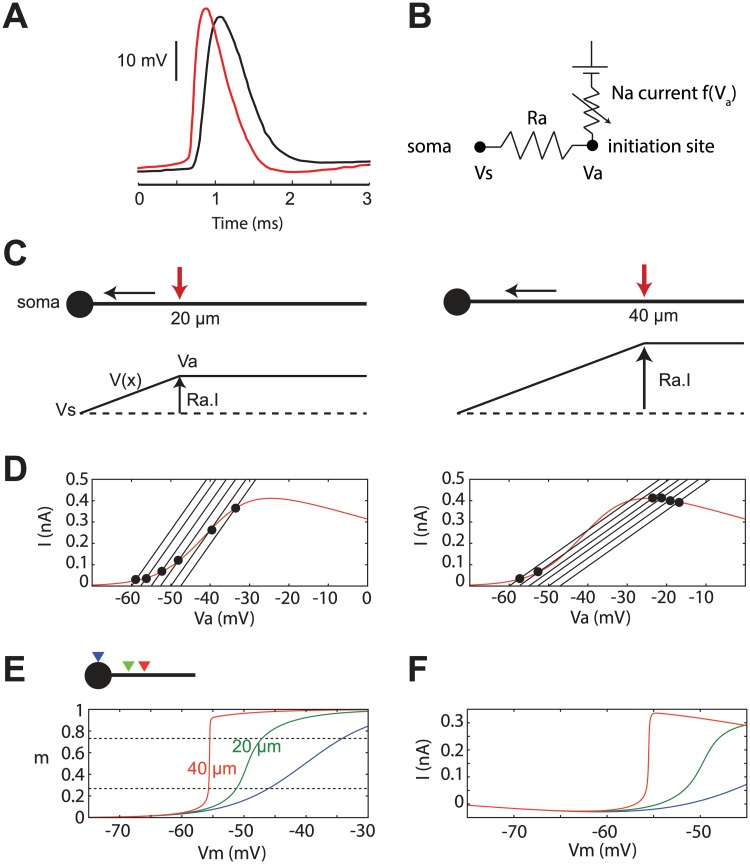
The compartmentalization hypothesis (B-F adapted from [31]). (A) Dual-patch recording of an action potential in soma (black) and in the axonal initial segment (red) (digitized from [41]). (B) Electrical model of the soma and initiation site (Vs: somatic voltage; Va: axonal voltage; Ra: axial resistance). (C) A current injected close to the soma produces a linear depolarization between soma and injection point (bottom: voltage as a function of distance to soma). The depolarization is proportional to Ra, and therefore to distance (left: injection at 20 μm; left: at 40 μm). (D) Na current (red curve) and axonal current (black lines) as a function of Va when Na channels are placed at 20 μm (left) and 40 μm (right) away from the soma. The 5 lines correspond to Vs between-60 mV and-47.5 mV. (E) Proportion of open Na channels as a function of Vs, with Na channels at the soma (dark blue), at 20 μm (green), and at 40 μm (red) away from the soma. (F) Current-voltage relationship measured at the soma (same color code as in E).

The third theoretical explanation, the compartmentalization hypothesis, is compatible with standard biophysics, and relies on the axonal initiation of spikes and on the fact that the soma acts as a current sink, because of its large size compared to the axon [31]. In cortical pyramidal cells, the initiation site is about 20–40 μm from the soma, while the electrotonic length of the axon is about 500 μm [45]. Thus, before spike initiation, voltage should be essentially constant across the AIS and follow the somatic voltage. However, at spike initiation, voltage varies by about 100 mV over 30 μm, so the neuron is not isopotential (Fig. 5A). This voltage gradient reflects a positive current flowing between the two sites, which is presumably the Na current (indeed applying tetrodotoxin (TTX) abolishes this gradient [46]). According to Ohm’s law, that current equals the voltage difference (100 mV) divided by the axial resistance (Ra) between the two sites. This is the axonal current measured in somatic voltage-clamp experiments at spike initiation (Fig. 3C) [20].

Why does this current flow abruptly when the somatic voltage exceeds a threshold, rather than gradually, as would be expected from the activation curve of Na channels? A simple electrical model shows how the phenomenon arises from the electrical coupling between the two sites (Fig. 5B). The spatial position of Na channels plays a critical role. When a current is injected at a point in the axon, most of the current flows to the soma—the soma is a current sink because it is much larger than the axon. Therefore there is a linear depolarization between the two sites, which equals the injected current times the axial resistance (Fig. 5C). This resistance is proportional to the distance between the two sites. Therefore, injecting the same current further away from the soma produces a proportionally larger depolarization. This injected current is the Na current, which depends on the voltage Va at the initiation site: I = f(Va). It must equal the axonal current, given by Ohm’s law: I = (Va-Vs)/Ra (Vs is the somatic voltage). Thus, the axonal voltage is determined by the electrical coupling identity: f(Va) = (Va-Vs)/Ra. This identity is represented in Fig. 5D, where the red curve is the Na current as a function of Va, and black lines are the axonal current as a function of Va, for different values of the Vs. The intersection of these curves determines I and Va. When the Vs is increased, both I and Va increase gradually, corresponding to the gradual opening of Na channels. But something interesting happens when channels are placed far from the soma, so that the axial resistance is large (Fig. 5D, right): when Vs is increased past some threshold value, the intersection point suddenly jumps to a much higher value (mathematically, a bifurcation). This means that Na channels open almost all at once when Vs exceeds a threshold voltage (Fig. 5E, red). Seen from the soma, this spatial coupling produces a sharpening of the current-voltage curve (Fig. 5F). This phenomenon happens when Na channels are placed above a critical distance from the soma. Phenomenologically, the neuron then behaves as an IF neuron, not as a single-compartment HH model. Note that this explanation only relies on an analysis of the Na current, because other channels expressed in the AIS have slower dynamics and are therefore not expected to be involved in the initiation of spikes (but possibly in the modulation of excitability [47]). Indeed the activation time constant of Na channels at physiological temperature near spike threshold is likely to be smaller than 100 μs [48].

## Conclusion

What is the most realistic single-compartment model of spike initiation? The single-compartment HH model has more empirical content than any model of the integrate-and-fire type: it also makes predictions about ionic currents and about spike shape. However, these predictions are falsified by empirical observations: spikes show a distinct kink at onset, and Na current varies discontinuously with voltage. This failure might not come as a surprise, since spikes are initiated in the axon and a single-compartment model does not take this fact into account. What is more surprising is that this fact actually makes the IF model more realistic than the single-compartment HH model (with channel properties obtained from patch-clamp measurements) in predicting the spiking responses of neurons (in particular at high input frequency). This increased realism may have some functional importance, because the sharp initiation of spikes allows neurons to phase lock to high frequency inputs and to quickly signal fast changes in input current [23].

It might be objected that the single-compartment HH model is actually realistic, if the compartment is meant to represent the initiation site (in the AIS) and not the soma. This is not correct. Such a model would indeed correctly predict some aspects of spike shape in the AIS (specifically, “onset rapidness” in phase plots [24,31]). But the model would still not account for the increased sharpness of spike initiation, in particular the transmission of high frequencies. In the compartmentalization hypothesis [31], it is the electrical coupling between AIS and soma that is responsible for the increased sharpness, not the sole fact that spikes are initiated in the axon. Another reason that the AIS is inadequate to represent the location of a single-compartment model is that the input to the AIS is not the total dendritic current coming from the synapses, but the axonal current flowing between the soma and AIS. That current is determined by the voltage gradient between the two sites, and therefore correct modeling of the input to the AIS would require two compartments.

This conclusion does not imply that the HH formalism is inadequate. In the compartmentalization hypothesis, a multicompartmental HH model can account for all aspects of spike initiation. Such a detailed model has more empirical content than an IF model and therefore is potentially more realistic, if properly adjusted. The cooperativity hypothesis is a more radical departure from standard biophysics. Although it is not supported by direct experimental evidence, it has also not been directly challenged in intact preparations (only in axonal blebs).

The conclusion also does not imply that the single-compartment HH model has no value. A pump is a valuable model of the heart, even though it is arguably not realistic. The single-compartment HH model has some explanatory value: it does explain correctly how Na currents create a positive feedback that yield to an explosion of the membrane potential. However, this conclusion does have important implications for scientists using single-compartment models in simulations or analysis: IF models are more realistic than HH or quadratic models.
